# Reduced expression of let‐7f activates TGF‐β/ALK5 pathway and leads to impaired ischaemia‐induced neovascularization after cigarette smoke exposure

**DOI:** 10.1111/jcmm.13144

**Published:** 2017-03-27

**Authors:** Wahiba Dhahri, Sylvie Dussault, Paola Haddad, Julie Turgeon, Sophie Tremblay, Kevin Rolland, Michel Desjarlais, Katia Y Cáceres‐Gorriti, Raphael Mathieu, Alain Rivard

**Affiliations:** ^1^ Department of Cardiovascular Research Centre Hospitalier de l'Université de Montréal Montréal Québec Canada

**Keywords:** neovascularization, angiogenesis, microRNA, let‐7f, TGF‐βR1, ALK5, smoking

## Abstract

This study sought to determine the potential role of microRNAs (miRNAs) in the detrimental effects of cigarette smoke on angiogenesis and neovascularization. Using large‐scale miRNA profiling and qRT‐PCR analyses, we identified let‐7f as a pro‐angiogenic miRNA which expression is significantly reduced in HUVECs treated with cigarette smoke extracts (CSE), and in the ischemic muscles of mice that are exposed to cigarette smoke (MES). In a mouse model of hindlimb ischaemia, intramuscular injection of let‐7f mimic restored ischaemia‐induced neovascularization in MES. Doppler flow ratios and capillary density in ischemic muscles were significantly improved in MES treated with let‐7f mimic. Clinically, this was associated with reduced ambulatory impairment and hindlimb ischaemic damage. Treatment with let‐7f mimic could also rescue pro‐angiogenic cell (PAC) number and function (attachment, proliferation, migration) in MES. ALK5 (TGF‐βR1), an important modulator of angiogenesis, is a target of let‐7f. Here we show that ALK5 is increased in HUVECs exposed to CSE and in the ischaemic muscles of MES. This is associated with a downstream activation of the anti‐angiogenic factors SMAD2/3 and PAI‐1. Importantly, treatment with let‐7f mimic reduces the expression of ALK5, SMAD2/3 and PAI‐1 both *in vitro* and *in vivo*. Moreover, let‐7f overexpression or ALK5 inhibition can rescue angiogenesis in HUVECs exposed to CSE. Cigarette smoke exposure is associated with reduced expression of let‐7f and activation of the anti‐angiogenic TGF‐β/ALK5 pathway. Overexpression of let‐7f using a miRNA mimic could constitute a novel therapeutic strategy to improve ischaemia‐induced neovascularization in pathological conditions.

## Introduction

After ischaemia, one of the most important physiological responses to maintain tissue integrity is the ability to develop new blood vessels (neovascularization) 1. Neovascularization is a complex process that necessitates the activation, the proliferation and the migration of mature endothelial cells in order to extend the pre‐existing vascular network (i.e. angiogenesis) 2. In addition to angiogenesis, it is now recognized that post‐natal neovascularization also depends on the action of bone marrow‐derived PACs 3, 4. PACs have been shown to reach sites of ischaemia where they promote neovascularization mainly through paracrine secretion of growth factors and cytokines 5.

Angiogenesis and neovascularization are dependent on important growth factors such as vascular endothelial growth factor (VEGF) 6, fibroblast growth factor (FGF) 7 and nitric oxide (NO) 8, 9. Members of the transforming growth factor‐β (TGF‐β) superfamily have also been shown to exert important functional effects in endothelial cells, and it has been proposed that these effects can vary depending on the context. TGF‐β signalling is initiated by ligand binding to a high‐affinity transmembrane TGF‐β type II receptor, which subsequently phosphorylates the TGF‐β type I receptor activin‐like kinase (ALK) 10. Recent studies have demonstrated that TGF‐β can bind 2 distinct type 1 receptors (TβRI), which have opposite effect on endothelial cell function and angiogenesis. Whereas ALK5 inhibits EC migration and proliferation, ALK1 promotes both processes after stimulation by TGF‐β1 11, 12.

It is now recognized that pathological conditions and risk factors involved in the development of atherosclerosis are also often associated with impaired neovascularization in response to ischaemia 1. Cigarette smoking is a major modifiable cardiovascular risk factor. In previous studies, we and others have shown that exposure to cigarette smoke leads to impaired angiogenesis, both *in vitro* 13 and *in vivo* 14, 15. In humans, cigarette smoking has also been associated with a reduction in the number and the functional activities of PACs 16, 17. However, the precise mechanisms responsible for the detrimental effects of cigarette smoke on vascular function and angiogenesis are still largely unknown.

MicroRNAs (miRNAs or miRs) represent a novel class of endogenous non‐coding small RNA molecules (20–25 nucleotides) that regulate a wide range of physiological and pathological processes, including angiogenesis 18, 19. The importance of mi‐RNAs as a group in angiogenesis and endothelial function was initially revealed by disrupting the function of Dicer and Drosha, two key enzymes for miRNA biogenesis 20, 21. Gene silencing of Dicer and Drosha in endothelial cells reduced the expression of lef‐7f and mir‐27b 21. Inhibitors against let‐7f and mir‐27b impaired endothelial cell tubule formation *in vitro*, indicating that these miRNAs promote angiogenesis by targeting anti‐angiogenic genes 21. Several other miRs have since been found to promote angiogenesis including miR‐126, miR‐130a and the miR‐17~92 cluster 18, 19. Collectively, these miRs have been referred to as pro‐angiomiRs 22. Here we suggested that impaired expression of pro‐angiomiR(s) could contribute to reduce neovascularization in pathological conditions. The present report shows for the first time that reduced expression of microRNA let‐7f contributes to impair angiogenesis, PAC function and ischaemia‐induced neovascularization following cigarette smoke exposure. We also demonstrate the potential role of the anti‐angiogenic TGF‐β/ALK5 pathway in that process.

## Materials and methods

### Cell culture

Human umbilical vein endothelial cells (HUVECs) were purchased from Life Technologies (Carlsbad, CA, USA) and cultured in medium 200 (Life technologies) supplemented with 8% foetal bovine serum (FBS, Wisent, St‐Jean‐Baptiste, QC, Canada), 100 IU/ml penicillin/0.1 mg/ml streptomycin (Wisent) and low serum growth supplement (LSGS; 2% FBS, 3 ng/ml bFGF, 10 mg/ml heparin, 1 mg/ml hydrocortisone and 10 ng/ml EGF; Life Technologies). In some experiments, HUVECs were treated with TGF‐β1 (10 nM, Sigma‐Aldrich) with or without the specific ALK5 inhibitor SB 431542 (2 μM, Sigma‐Aldrich). Cells were grown at 37°C, 5% CO_2_ and 95% air, and the medium was changed every 2 days. HUVECs were passaged when they reached 90% confluence and passages 4–6 were used for all experiments.

### Preparation of CSE

CSE was prepared as previously described 14. Briefly, two cigarettes were combusted with a modified syringe‐driven apparatus. The smoke was bubbled through 50 ml of medium 200 at a speed of 4 min. per cigarette. The resulting suspension was filtered through a 0.20‐μm pore filter (Millipore Corporation, Bedford, MA, USA) to remove bacteria and large particles. The filtered CSE was shown to be free of endotoxin (Gel clot LAL, Lonza, Switzerland). CSE was applied to HUVECs in culture within 30 min. of preparation to obtain a final concentration of 10%. This concentration corresponds approximately to exposure associated with smoking 1.5 pack per day, and was chosen based on previous reports showing physiological responses at this level 14.

### RNA isolation, microRNA array profiling and target prediction software analysis

Enriched low molecular weight (LMW) RNA was extracted from cultured HUVECs exposed or not to 10% CSE using the Ambion mirVana™ miRNA isolation kit (Life Technologies) according to the manufacturer's protocol. Microarray experiment was performed using the GeneChip miRNA v.1.0 array (Affymetrix, Santa Clara, CA, USA) which contains 46228 probes comprising 7815 probe sets and covering 71 organisms. Among the 7815 probe sets, 847 correspond to human miRNAs and another 922 to human snoRNAs and scaRNAs; 250 ng of LMW RNA was labelled with FlashTag™ Biotin HSR RNA Labeling Kit (Genisphere, Hatfield, PA, USA) according to the manufacturer's recommendations. A colorimetric ELOSA (enzyme linked oligosorbent assay) was performed to confirm the success of the biotin labelling process. The labelled samples were hybridized onto the arrays for 16 hrs at 48°C. The arrays were then washed, stained and scanned according to the manufacturer's instructions (Affymetrix). The Affymetrix GeneChip^®^ system was controlled by the GeneChip^®^ Command Console (AGCC) software. The raw data files of each sample were imported into the Affymetrix miRNA QC Tool software to normalize the data and to assess the quality control. The data were normalized by Robust Multichip Average (RMA) algorithm, which uses background adjustment, quantile normalization and summarization. Potential targets of let‐7f were identified with the miRWalk 2.0 database 23 which gathers the information produced by 12 existing miR‐target prediction programs (DIANA‐microTv4.0, DIANA‐microT‐CDS, miRanda‐rel2010, mirBridge, miRDB4.0, miRmap, miRNAMap, doRiNA, i.e. PicTar2, PITA, RNA22v2, RNAhybrid2.1 and Targetscan6.2).

### qRT‐PCR evaluation of miRNA expression

About 50 ng of total RNA was reverse‐transcribed using the TaqMan^®^ MicroRNA Reverse Transcription Kit (Life Technologies) as described by the manufacturer. Before use, RT samples were diluted 1:5. Gene expression level was determined using TaqMan MicroRNA assays (Cat. # 4427975, Life Technologies). qPCRs were performed using 1–5 ng of cDNA samples, using Perfecta qPCR Fastmix II (Quanta) and 2 μM of each primer. The Viia7 qPCR instrument (Life Technologies) was used to detect the amplification level and was programmed with an initial step of 20 sec. at 95°C, followed by 40 cycles of 1 sec. at 95°C and 20 sec. at 60°C. Relative expression (RQ = 2^−ΔΔCT^) was calculated using the Expression Suite software (Life Technologies), and normalization was performed using both U6snRNA and SnoRNA202.

### miRNA transfection in HUVECs

Transfections were carried out at a concentration of 24 nM using Lipofectamine RNAiMAX Reagent (Life Technologies) according to the manufacturer's protocol as previously described 24. HUVECs were transfected 24 hrs after being plated in 6‐well plates with the following miRs purchased from Dharmacon (GE Healthcare Dharmacon, Lafayette, CO, USA): miRIDIAN miR mimic negative control #1, miRIDIAN miR mimic hsa‐let‐7f‐5p and miRIDIAN hsa‐let‐7f Hairpin Inhibitor. After 24 hrs, the transfection medium was replaced with antibiotic‐free complete M200 medium and cells were exposed or not to CSE 4 hrs later. Transfection efficiency was measured using a mimic transfection control Dy547 (Dharmacon) and found to be around 80%.

### HUVEC network formation on Matrigel and cell migration

The angiogenic activity of HUVECs was determined using the Matrigel network formation assay as previously described 13. Briefly, after transfection and CSE exposure, HUVECs were plated at a density of 20 000 cells/well in 96‐well plates precoated with 50 μl of growth factor reduced Matrigel Matrix (Becton Dickinson Labware, Bedford, MA, USA) and cultured at 37°C for 6 hrs in presence of 50 ng/mL of VEGF. Capillary‐like branches were observed under a light microscope. Images were obtained at a 50× magnification, and all branches were counted. Cell migration was assessed using a modified Boyden chamber assay as previously described 13.

### 3D angiogenesis assay (spheroid assay)

Generation of HUVEC spheroids and angiogenesis assays was performed as previously described 25. Briefly, 750 transfected cells were cultured in complete M200 medium containing 0.25% (w/v) methylcellulose as 20 ul hanging drops for 24 hrs. 100–125 collected spheroids were suspended in 375 μl of complete M200 medium containing 0.5% methylcellulose and embedded into collagen gels (8 volumes of home‐made rat tail collagen, 1 volume of 10× DMEM and 1 volume of NaOH). The spheroid‐containing gel was allowed to solidify for 30 min. before 100 μl of basal medium containing 500 ng/ml of VEGF with or without CSE was added on top of the gel. All plates were incubated for 48 hrs at 37°C, 5% CO_2_ and 100% humidity. Total sprout length was than measured using Metamorph software in 10 spheroids per condition.

### Wound healing (scratch assay)

Endothelial cell wound healing was assessed by an adapted scratch assay in confluent HUVECs as previously described 26. The cells were transfected and grown to near confluence in 24‐well plates and exposed or not to 10% CSE for 16 hrs. Mechanical disruption of the monolayer was realized by scraping with a pipette tip. The cells were then stained with crystal violet for 10 min. at room temperature. The number of cells covering the wound was quantified using an inverted microscope at a magnification of 200× by an investigator blinded to the experimental conditions. Three fields per well were evaluated, and all experiments were performed in duplicate.

### Exposure of mice to cigarette smoke

The protocol was approved by the Comité Institutionnel de Protection des Animaux (CIPA) of the Centre Hospitalier de l'Université de Montréal (CHUM). 6–8 week‐old female C57BL/6 mice were purchased from Charles River (St‐Constant, QC, Canada). Mice were exposed to cigarette smoke (two cigarettes, twice a day) *via* smoking machine as we previously described 14 starting 14 days prior to surgery until killing Commercial cigarettes (Player's Plain, tar: 17 mg, nicotine: 1.5 mg, carbon monoxide: 12 mg) were used. The mice appeared normal during the entire experimental period. Control mice were restrained in an identical cage for the same period of time but were not exposed to cigarette smoke.

### Murine ischaemic hindlimb model

Unilateral hindlimb ischaemia was surgically induced after anaesthesia with 2% isoflurane as previously described 27. The model we use is the one originally described by Coufinhal *et al*. 28. It involves complete removal of the femoral artery together with all major collateral branches. As reviewed by Limbourg *et al*., although this model precludes analysis of arteriogenesis, it is associated with more ischaemia and more ischaemia‐induced angiogenesis compared to a model using only femoral artery ligation 29. At the time of surgery, mice were injected intramuscularly with 5 mg/kg of *in vivo* ready mirVana^®^ miRNA mimic mmu‐Let‐7f‐5p, or mirVana^®^ miRNA mimic negative control #1 (Life technologies). This dose was chosen based on preliminary experiments showing optimal transfection efficiency and Let‐7f upregulation in muscles. miRNAs were administered in a solution of Max suppressor RNA‐LANCEr II (Bioo Scientific, Austin, TX, USA) according to the manufacturer's recommendations. Ambulatory impairment was evaluated using a scale from 1 (normal walking) to 4 (walking with the leg dragging behind) 30. Evaluation of the ischaemic damage of the leg and foot was evaluated using a scale from 0 (no necrosis) to 4 (amputation) 30. The mice were killed at predetermined arbitrary time‐points after surgery with an overdose of sodium pentobarbital.

### Monitoring of blood flow

Hindlimb blood flow (12–15 mice/group) was monitored with a laser Doppler perfusion imager (LDPI) system (Moor Instrument Ltd., Axminster, UK) after anaesthesia with a ketamine–dexmedetomidine solution (50 mg/kg and 0.5 mg/kg, IP) 27. Measurements were performed in the supine position with the legs lying flat on the surgical carpet. The legs were put at a 90‐degree angle at the heel to achieve similar degrees of rotation. The region of interest was the distal part of the leg (including the foot), and data acquisition was performed from the knee down to the tip of the toes. Laser Doppler analyses were performed by a single observer blinded to the treatment group at days 0, 3, 7 and 21 after surgery. After LDPI measurements, dexmedetomidine was antagonized with a solution of atipamezole (1 mg/kg, SC). To account for variables such as ambient light and temperature, the results are expressed as the ratio of perfusion in the left (ischaemic) *versus* right (non‐ischaemic) hindlimb.

### CD31 immunohistochemistry

Whole ischaemic hindlimbs were harvested 21 days after surgery and immediately fixed in Tissufix (Chaptec, Montreal, QC, Canada) overnight. After bones were carefully removed, 3‐mm‐thick tissue transverse sections of the hindlimbs were cut at the level of the gastrocnemius muscle and paraffin‐embedded so that the whole leg could be analysed on each section. Identification of endothelial cells was performed by immunohistochemistry for CD31 with a rat monoclonal antibody directed against mouse CD31 (BD Pharmigen, San Diego, CA, USA). Capillaries were counted by a single observer blinded to the treatment regimen at a 200× magnification. Results were expressed as capillary density per field 27.

### PACs isolation and characterization

Seven days after hindlimb ischaemia, mouse bone marrow mononuclear cells were isolated from the femora and tibiae by flushing the bone marrow cavities using culture medium 27, and kept on fibronectin‐coated plates (Sigma‐Aldrich, St. Louis, MO, USA). After 4 days in culture, non‐adherent cells were removed by thorough washing with PBS. Adherent cells were stained with DAPI (0.5 mg/ml; Life Technologies), 1,10‐dictadecyl‐3,3,30,30 tetramethylindocarbocyanine perchlorate acetylated low‐density lipoprotein (DiI‐acLDL, 2.5 mg/ml for 1 hr, Life Technologies) and FITC‐labelled lectin BS‐1 (Bandeiraea simplicifolia, 10 mg/ml for 1 hr, Sigma‐Aldrich, St‐Louis, MO, USA). PACs, also known as ‘early outgrowth EPCs’, can express endothelial markers but also myeloid markers such as CD45 and CD14, attesting for a probable monocytic origin. In our experiments, spindle‐shaped cells were observed, and the vast majority of adherent cells (95%) were found to be double positive for the uptake of DiI‐labelled acetylated LDL and binding of FITC‐labelled lectin. Characterization of our mouse bone marrow PAC population by FACS analysis indicates that 83% of adherent cells express CD45, 57% CD14, 18% CXCR4, 9% CD31 and 8% Sca‐1. These values are similar to previous studies in mice and consistent with a probable monocytic origin. We also found that these cells can migrate in response to VEGF stimulation and are capable of incorporating into a network of tubular‐like structures when cocultured with mature endothelial cells. On the basis of these morphological and functional characteristics and in line with previous studies, these cells were characterized and referred to in the manuscript as PACs.

### PAC adhesion to an endothelial monolayer

A monolayer of HUVECs (passage 4–6) was prepared in 24‐well plates. HUVECs were pretreated for 16 hrs with tumour necrosis factor‐a (1 ng/ml; BD Biosciences, San Jose, CA, USA), fixed and stained with DAPI (0.5 mg/ml; Life Technologies). PACs were labelled with DiI‐AcLDL and 15 000 cells were added to each well (2 wells/mouse) and incubated for 3 hrs at 37°C. Non‐attached cells were gently removed with PBS and adherent PACs were fixed with 2% paraformaldehyde and counted in three random fields per well 27.

### PACs migration assay

PAC migration was assessed using a modified Boyden chamber assay 27; 15 000 cells in growth factor deprived medium were added to the upper chamber of a transwell insert (pore size 8 μm; Corning, Corning, NY, USA) coated with 0.1% gelatin. The inserts were placed in a 24‐well plate containing medium 200 with 50 ng/ml VEGF. After incubation for 6 hrs at 37°C, the cells which did not migrate were removed by wiping the upper surface with an absorbent tip. Migrated cells were fixed for 10 min. with 3.7% formaldehyde and stained with haematoxylin. The number of cells that had migrated was counted in three different representative high‐power (200x) fields per insert. All experiments were performed in duplicate.

### Proliferation assay

PAC proliferation was assessed using the MTS Celltiter 96 aqueous non‐radioactive cell proliferation assay (Promega, Madison, WI, USA) as previously described 13. After 4 days in culture, 15 000 PACs were plated in 96‐well plates coated with 1% gelatin. Once cells were attached, they were incubated for 24 hrs with 50 ng/ml of VEGF. After the treatments, MTS was added to each well to achieve final concentrations of 0.04 mg/ml. PAC proliferation was quantified after 4 hrs by densitometric analysis of MTS tetrazolium compound. Optical density was recorded with a microplate reader at 490 nm. Readings were corrected for background optical density by subtracting the readings from M200/MTS incubated at the same time in the absence of PACs. The results from each group of mice performed in triplicate are represented.

### Western blot analysis

Protein levels were analysed by Western blots in ischaemic muscles homogenates and in HUVEC extracts. For total protein extraction, isolated muscles from whole hindlimbs were rinsed in PBS to remove excess blood, snap‐frozen in liquid nitrogen and stored at −80°C until use. Whole‐cell protein extracts were obtained after homogenization of ischaemic muscles of the different groups of mice in ice‐cold RIPA buffer (pH = 8) containing 50 mM Tris–HCl, 150 mM NaCl, 5 mM EDTA, 1% Triton X‐100, 0.5% sodium deoxycholate, 0.1% SDS with a cocktail of proteases and phosphatase inhibitors (MiniComplete, PhosphoStop and PMSF, Roche, Bâle, Switzerland). Transfected HUVECs were lysed with 150 μl of RIPA lysis buffer, harvested and sonicated. 50 ug of protein per muscle homogenate sample and 25 ug of protein per cells lysates sample were separated on an SDS‐polyacrylamide gel and electroblotted on nitrocellulose membranes. Non‐specific binding sites were blocked with 5% skim milk powder in TBS‐T (50 mM Tris–HCl, 140 mM NaCl, 0.05% Tween‐20) for 1 hr. The membranes were probed overnight at 4°C with the following antibodies: ALK5 (TGFβR‐I, 1:1000; Cell Signaling Technology, Danvers, MA, USA), Phospho‐Smad2 (Ser465/467)/Smad3 (Ser423/425); 1:1000; Cell Signaling Technology), Phospho‐SMAD1/5 (Ser‐463/465; 1:1000; Cell Signaling Technology), PAI‐1 (1:1000; Santa Cruz Biotechnology, Dallas, TX, USA) or β‐actin (1:1000, Santa Cruz Biotechnology). Membranes were then washed three times for 10 min. with TBS‐T and incubated with anti‐rabbit secondary antibodies conjugated with HRP (1:3000) for 1 hr and washed with TBS‐T. Specific proteins were detected by chemiluminescent reaction (GE Healthcare, Piscataway, NJ, USA) followed by exposure to Hyperfilm ECL (GE Healthcare). Protein expression was quantified using ImageJ, and the results are expressed as density values normalized to the loading control (β‐actin).

### Statistical analysis

Statistical significance was evaluated by anova followed by a Bonferonni *post hoc* test. For the laser Doppler measurements, a two‐way repeated‐measures anova with Bonferroni *post hoc* test was used. For categorical variables, a chi‐square test was used. A value of *P* < 0.05 was interpreted to denote statistical significance.

## Results

### Effect of cigarette smoke exposure on miR expression

**Figure 1:**
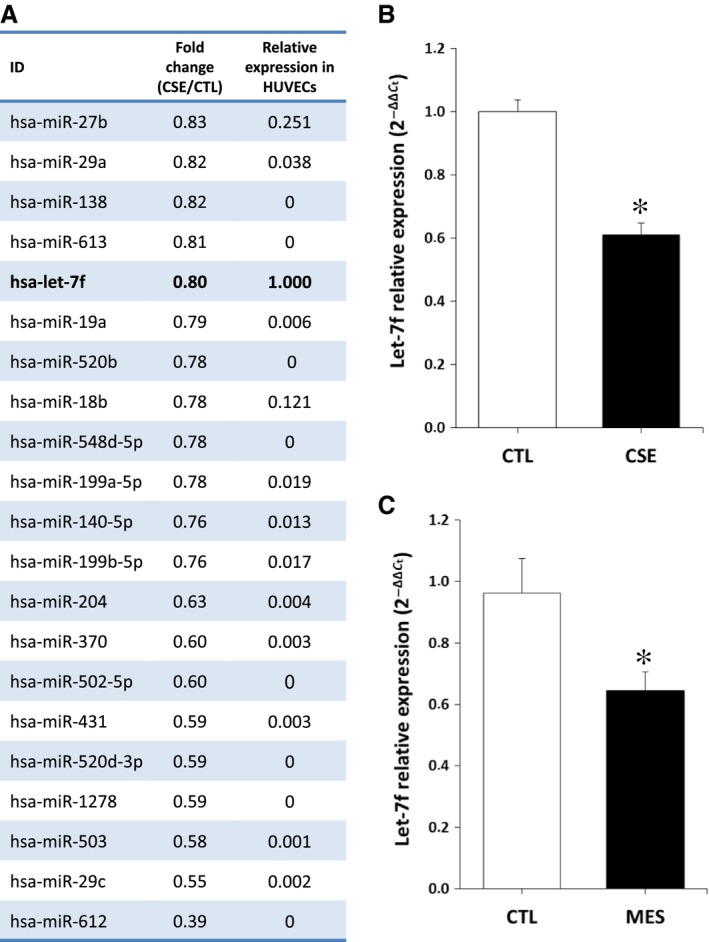
Effect of cigarette smoke exposure on miRNA expression. **A**. List of 21 miRs that were down‐regulated by more than 15% in HUVECs exposed to CSE, as assessed by Affymetrix GeneChip array analysis (n = 3/group). **B**–**C.** Relative expression of miR let‐7f in HUVECs exposed to CSE (n = 5/group) (**B**) and in the ischaemic muscles of MES, (**C**) as quantified by real‐time qPCR (n = 8/group). Data are mean ± S.E.M.*P < 0.05 *versus* CTL.

We used Affimetrix microarray analyses to identify potential angiomiRs that could be impaired in HUVECs exposed to CSE. Among 21 miRs that were modulated by at least 15% in our microarray (CSE *versus* CTL), microRNA Let‐7f had by far the highest expression in HUVECs. Other miRs were weakly expressed compared with Let‐7f and several were even undetectable (Fig. 1A). Interestingly, let‐7f has been shown to have pro‐angiogenic effects in endothelial cells 21. Moreover, let‐7f was previously found to be reduced in the lungs of rats exposed to cigarette smoke 31. Using RT‐PCR, we confirmed that the expression of let‐7f is significantly reduced in HUVECs exposed to CSE (39% reduction, Fig. 1B). Moreover, let‐7f is also significantly reduced in the ischaemic muscles (35% reduction, Fig. 1C), but not in the non‐ischaemic muscles (Fig. S1) of mice exposed to cigarette smoke. Therefore, in the following experiments, we focused on characterizing the specific role of let‐7f in the modulation of angiogenesis and neovascularization following cigarette smoke exposure.

### Let‐7f rescues cigarette smoke‐induced impairment of angiogenesis in endothelial cells

**Figure 2:**
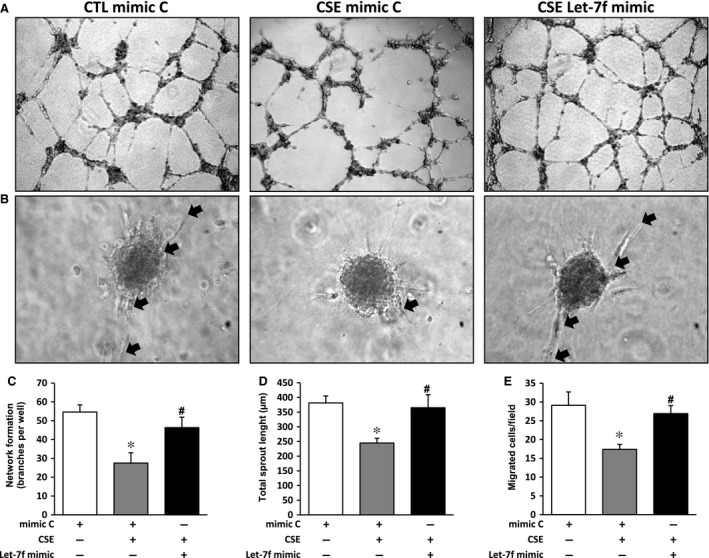
Effect of cigarette smoke exposure and let‐7f treatment on angiogenesis in endothelial cells. **A**–**D.** Evaluation of angiogenesis *in vitro* using a Matrigel assay (**A** and **C**, n = 8/group) and a spheroid assay (**B** and **D**, n = 4/group) in HUVECs exposed or not to CSE and treated with let‐7f mimic or a mimic control (mimic C). **E.** Evaluation of cell migration *in vitro* using a modified Boyden chamber assay in HUVECs exposed or not to CSE and treated with let‐7f mimic or a mimic control (mimic C) (n = 3/group). Data are mean ± S.E.M. *P < 0.05 *versus* mimic C; #*P* < 0.05 *versus* CSE+mimic C.

To investigate the role of let‐7f *in vitro*, we transfected HUVECs with a let‐7f mimic. Transfection efficiency was established to be more than 80%, as assessed using a labelled miR mimic (Fig. S2A–B). The level of Let‐7f was also significantly increased in transfected HUVECs, as assessed by qRT‐PCR (Fig. S2C). As seen in Figure 2, exposure to CSE significantly reduced network formation (Fig. 2A and C) and angiogenic sprouting from spheroids (Fig. 2B and D) in HUVECs. However, treatment with let‐7f completely restored network formation and angiogenic sprouting from spheroids in HUVECs exposed to CSE. In addition, let‐7f rescued endothelial cell migration in HUVECs exposed to CSE (Fig. 2E). On the other hand, treatment with a let‐7f inhibitor reduced network formation and migration activity in control HUVECs that had not been exposed to CSE (Fig S3).

### Effect of let‐7f on ischaemia‐induced neovascularization

**Figure 3:**
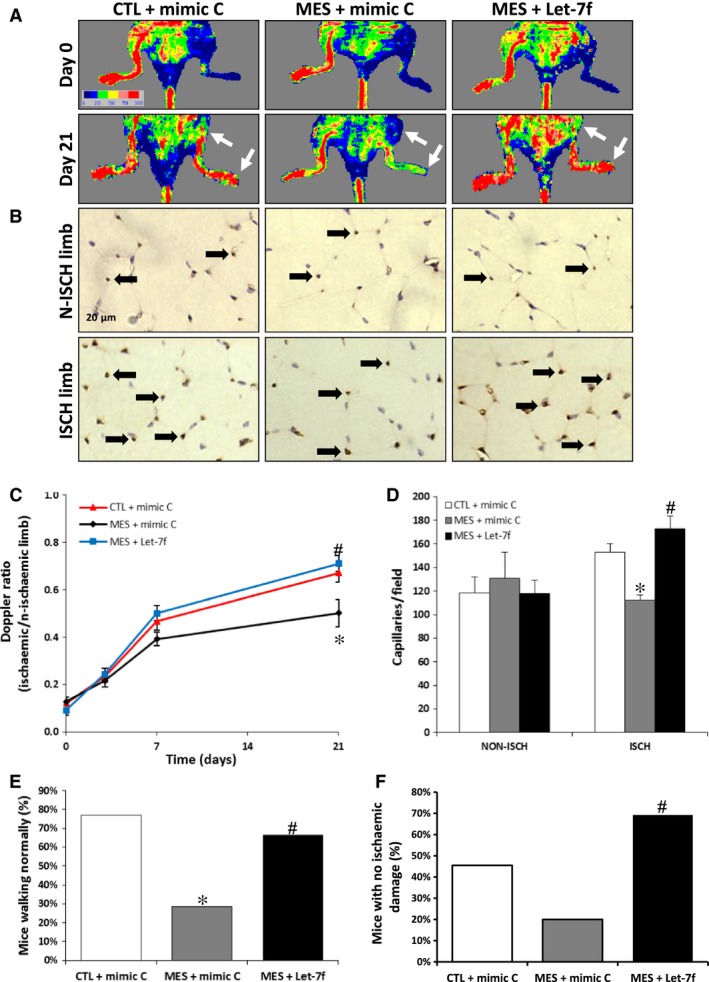
Effect of cigarette smoke exposure and let‐7f treatment on ischaemia‐induced neovascularization. **A** and **C.** Representative images (**A**) and quantification (**C**) of laser Doppler measurements after hindlimb ischaemia in control mice (CTL) and in MES treated with let‐7f or a scrambled miR control (mimic C). A colour scale illustrates blood flow variations from minimal (dark blue) to maximal (red) values. Arrows indicate region of interest in left ischaemic hindlimbs (n = 10–12/group). **B** and **D.** Representative images (**B**) and quantification (**D**) of CD31 immunostaining in ischaemic (isch) and non‐ischaemic (n‐isch) hindlimb muscles of the different groups of mice (n = 12–13/group). **E**–**F.** Percentage of mice walking normally (**E**) and presenting no ischaemic damage (**F**) in the different groups at day 21 after ischaemia (n = 11–15/group). *P < 0.05 *versus* CTL+mimic C; #*P* < 0.05 *versus* MES+mimic C.

Hindlimb perfusion was evaluated after surgically induced ischaemia by serial LDPI studies (Fig. 3A and C). At the time of surgery, mice were injected intramuscularly with a let‐7f mimic or a mimic negative control (mimic C). qRT‐PCR analyses confirmed that the expression of let‐7f was significantly increased in the ischaemic hindlimb muscles at day 3 after transfection with let‐7f mimic (Fig. S2D). Cigarette smoke exposure was associated with a significant impairment of blood flow recuperation at day 21 after surgery (Doppler flow ratio (DFR) 0.50 ± 0.03 *versus* 0.67 ± 0.04; *P* < 0.05). However, mice exposed to cigarette smoke (MES) but also treated intramuscularly with a let‐7f mimic showed an important improvement of perfusion at day 21 (DFR 0.71 ± 0.06 *versus* 0.50 ± 0.03; *P* < 0.05) and were therefore completely protected against cigarette smoke‐induced impairment of blood flow recuperation (Fig. 3C). A similar effect was observed at the microvascular level (Fig. 3B and D). Cigarette smoke exposure was associated with a significant decrease of capillary density in ischaemic muscles at day 21 after surgery (112 ± 5 *versus* 153 ± 7 capillaries/field; *P* < 0.05). However, MES treated with let‐7f mimic were protected against reduction of capillary density in ischaemic muscles (Fig. 3D). Clinically, the improvement of blood flow in animals treated with let‐7f was associated with a significant reduction of ambulatory impairment (Fig. 3E) and ischaemic damage (Fig. 3F) compared to MES treated with a mimic negative control.

### Let‐7f rescues the number and the functional activities of PACs

**Figure 4:**
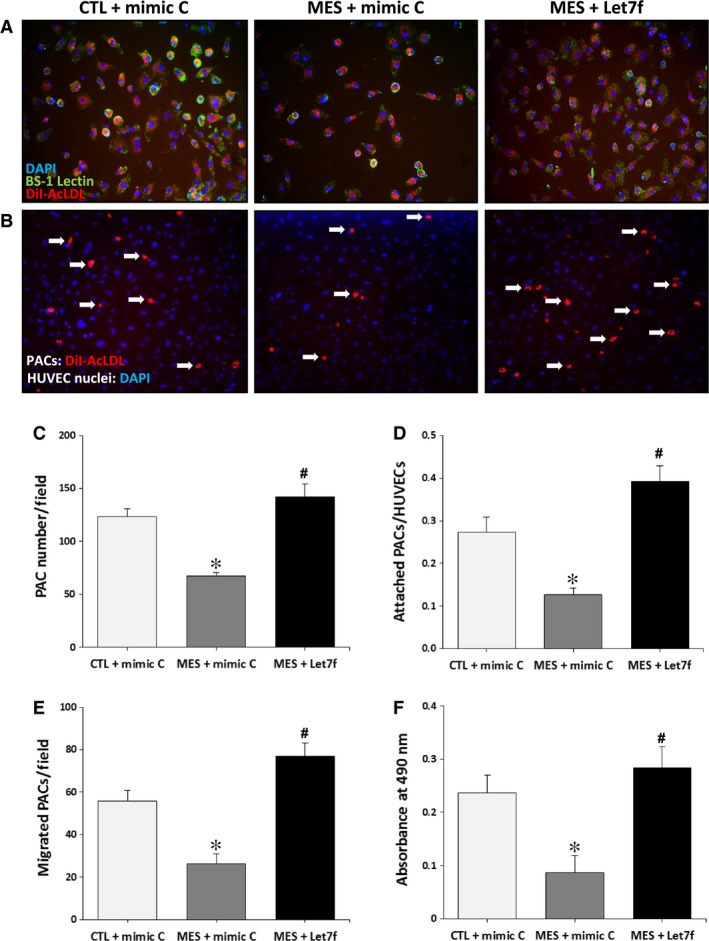
Effect of cigarette smoke exposure and let‐7f treatment on PAC number and function. **A** and **C.** Representative pictures (**A**) and quantification (**C**) of triple‐stained PACs (DAPI, BS‐1 lectin‐FITC and DiI‐acLDL) in control mice (CTL) and in MES treated with let‐7f or a scrambled miR control (mimic C). **B** and **D.** Representative pictures (**B**) and quantification (**D**) of PACs (red) adhesion to HUVECs (blue) in the different groups. **E**–**F.** VEGF‐induced migration (**E**) was assessed using a modified Boyden chamber assay and proliferation (**F**) was measured using an MTS assay. Data are mean ± S.E.M. (n = 4/group). *P < 0.05 *versus* CTL+mimic C; #*P* < 0.05 *versus* MES+mimic C.

PACs have been shown to reach sites of neovascularization where they can contribute to the formation of new blood vessels 5. However, the number and the functional activities of PACs are impaired by smoking 16, 17. Here we found that the number of PACs in the bone marrow was significantly reduced in mice exposed to cigarette smoke (Fig. 4A and C). Moreover, the functional activities of PACs including attachment to endothelial cells (Fig. 4B and D), migration (Fig. 4E) and proliferation (Fig. 4F) were impaired in mice exposed to cigarette smoke. However, treatment with let‐7f restored the number and the functional activities of PACs in mice exposed to cigarette smoke (Fig. 4A–F).

### Let‐7f and the TGF‐βR1/ALK5 pathway

**Figure 5:**
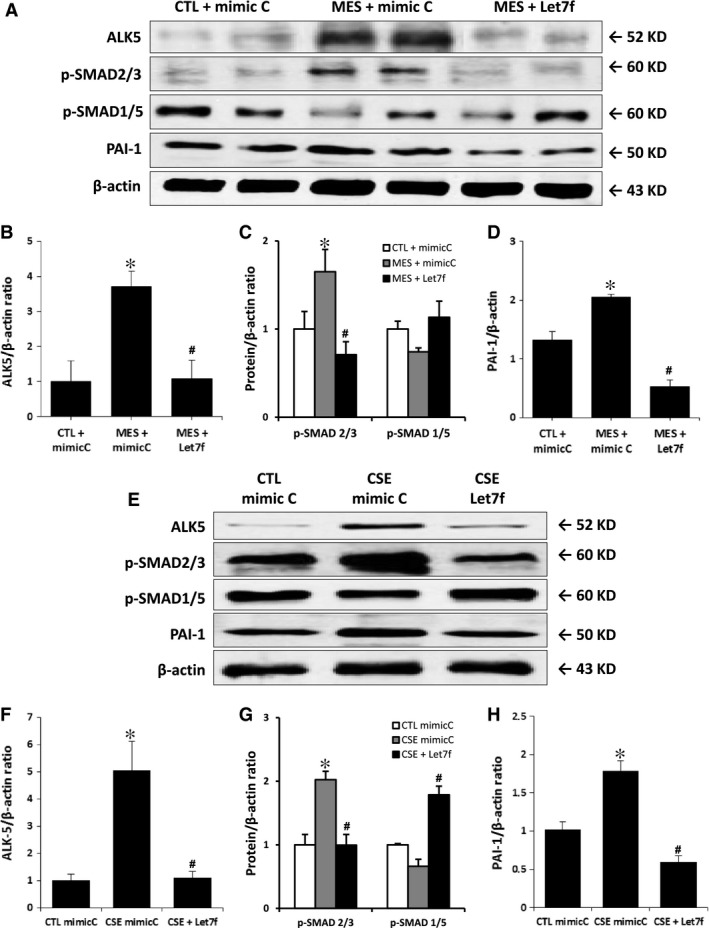
Effect of cigarette smoke exposure and Let‐7f treatment on TGF‐βR1/ALK5 pathway. Representative images and quantification of Western blots of ALK5, p‐SMAD2/3, p‐SMAD1/5 and PAI‐1 in the ischaemic muscles of mice (**A**–**D**) and in HUVECs (**E**–**H**) after different treatments. Protein expression was normalized to β‐actin. Data are mean ± S.E.M. n = 4/group (mice) and n = 3/group (HUVECs). *P < 0.05 *versus* CTL+mimic C, #*P* < 0.05 *versus* MES+ mimic C or CSE+mimic C.

**Figure 6:**
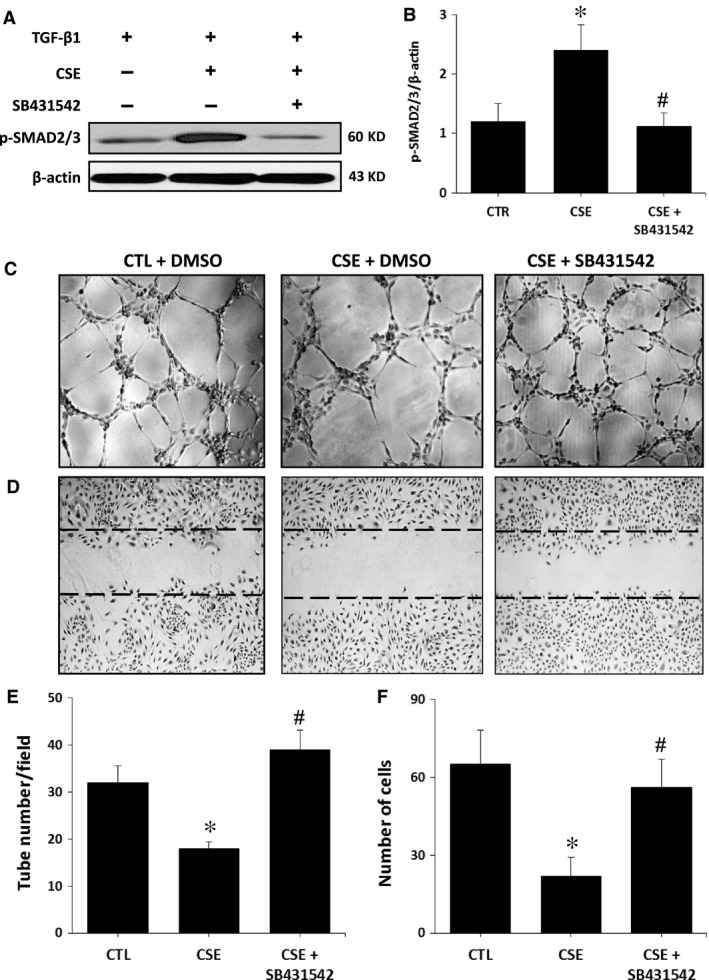
Effect of ALK‐5 inhibition on HUVEC functions. Representative Western blot (**A**) and quantification (**B**) of p‐SMAD2/3 in HUVECs exposed or not to CSE and treated or not with ALK5 inhibitor SB431542. **C**–**F**. Representative images and quantification of Matrigel assays (**C** and **E**) and scratch assays (**D** and **F**) in the different treatment groups. Data are mean ± S.E.M. (n = 3–4/group). *P < 0.05 *versus* CTL; #*P* < 0.05 *versus* CSE.

TGF‐βR1/ALK5 is one of the genes that are targeted by let‐7 miRs 23, 32. Stimulation of TGF‐βR1/ALK5 has previously been associated with inhibition of angiogenesis *via* SMAD2/3 and PAI‐1 activation 11. Here we found that ALK5 expression is significantly increased in the ischaemic muscles of mice exposed to cigarette smoke (Fig. 5A–B). This is associated with a robust activation of SMAD2/3 (but not SMAD1/5) and a downstream increase in the levels of PAI‐1. Interestingly, activation of the TGF‐β/ALK5 pathway by cigarette smoke is not seen in mice treated with a let‐7f mimic (Fig. 5A–D). Similar findings are observed in HUVECs where exposure to CSE leads to increased expression of ALK5, pSMAD2/3 and PAI‐1 that is preventable by let‐7f mimic treatment (Fig. 5E–H). In addition, treatment with the ALK5 inhibitor SB431542 reduces pSMAD2/3 activation and can restore the angiogenic activities (network formation, wound healing) of HUVECs exposed to CSE (Fig. 6).

## Discussion

To our knowledge, the present study is the first documentation of the essential role of let‐7f for angiogenesis and ischaemia‐induced neovascularization in a pathological situation. Although miRNAs are increasingly recognized as important factors involved in a variety of physiological conditions, their specific role for the modulation of neovascularization following tissue ischaemia remains largely unexplored. More specifically, the effect of different cardiovascular risk factors on the expression levels of miRNAs, and how this might influence the angiogenic response following ischaemia is currently unknown. Several cardiovascular risk factors have been shown to impair neovessel formation in response to ischaemia. These pathological conditions might at least in part explain the lack of efficacy of pro‐angiogenic therapies in atherosclerotic patients, compared with the positive results obtained in young and healthy animals 1. Cigarette smoking is one of the most important preventable risk factors leading to the development of atherosclerosis, acute cardiovascular events and premature deaths 33. Cigarette smoke exposure has also been associated with impaired neovascularization and blood flow recuperation after hindlimb ischaemia 14. Moreover, the number and the functional activities of PACs are reduced in both humans 16, 17 and animals 34, 35 that are exposed to cigarette smoke. However, the specific mechanisms that are involved in that physiopathology are not completely understood. The present study uncovered a novel mechanism by which modulation of miRNA expression could contribute to inhibit ischaemia‐dependent reparative responses in pathological conditions. It demonstrates for the first time that reduced expression of let‐7f following exposure to cigarette smoke leads to impairment of neovascularization and blood flow recuperation after tissue ischaemia.

miRNAs of the let‐7 family were initially described as important developmental molecules in *C*. *elegans*. Mature let‐7 family members are highly conserved across animal species and these miRNAs are known as key regulators of cell proliferation and differentiation 36. Let‐7 family members are often reported as tumour suppressors in humans. However, in the vascular system, members of the let‐7 family such as let‐7f and let‐7g have been associated with endothelial cell proliferation and angiogenesis. For instance, inhibitors of let‐7f have been shown to reduce sprout formation in endothelial cells, suggesting that let‐7f might promote angiogenesis by targeting anti‐angiogenic genes 21. Let‐7g was also recently shown to promote angiogenesis *in vitro* and to reduce endothelial cell senescence 32. Here we found that let‐7f is highly expressed in endothelial cells and that its expression is reduced both in endothelial cells treated with CSE and in the ischaemic muscles of mice that have been exposed to cigarette smoke. We used both gain‐ and loss‐of‐function approaches *in vitro* to demonstrate the role of let‐7f in the modulation of endothelial cell migration and network formation that is induced by CSE. Supplementation of let‐7f with a miR mimic could restore the angiogenic properties of endothelial cells exposed to CSE, whereas inhibition of let‐7f with an anti‐miR inhibited angiogenesis. Importantly, our *in vivo* study demonstrates that let‐7f supplementation using a miR mimic could have important therapeutic effects in the setting of ischaemia. Animals exposed to cigarette smoke and treated with let‐7f mimic demonstrated increased blood flow recovery, reduced ischaemic damages and improved mobility compared with animals exposed to cigarette smoke and treated with a mimic control. At the microvascular level, this was associated with increased capillary density in ischaemic muscles of let‐7f‐treated animals. The mechanism by which cigarette smoke reduces let‐7f expression is currently unknown. Interestingly, let‐7 is a hypoxia‐sensitive miRNA induced by HIFα 37 and we have previously shown that HIFα expression is reduced following cigarette smoke exposure 14. It is therefore possible that HIFα is involved in the inhibition of Let‐7f by cigarette smoke. In addition to the specific mechanism regulating Let‐7f expression, the exact components in cigarette smoke that are involved in the modulation of let‐7f and angiogenesis are also currently unknown. Cigarette smoke contains more than 4000 different chemicals 33. Future studies are needed to determine how specific compounds such as nicotine, polycyclic aromatic hydrocarbons or carbon monoxide might modulate miRNA expression and angiogenesis in ischaemic conditions.

In the present study, we propose that one of the mechanism by which let‐7f inhibition contributes to impair angiogenesis and neovascularization following cigarette smoke exposure involves the TGF‐β pathway. TGF‐β has an important role during vascular remodelling. It is classically perceived as an inhibitor of angiogenesis, being involved in the resolution phase of this physiological process 38. In most cell types, TGF‐β acts through activation of TGF‐βR1, also known as ALK5. Endothelial cells also express ALK1, another TGF‐β type 1 receptor. It has been proposed that the balance of activation between these two receptors could contribute to explain the pro‐ or anti‐angiogenic effects of TGF‐β in different contexts *in vivo* 11. TGF‐βR1/ALK5 is targeted by let‐7 miRs 23, 32. Here we found that reduced expression of let‐7f following cigarette smoke exposure correlated with increased expression of ALK5, both in endothelial cells exposed to CSE and in the ischaemic muscles of MES. ALK5 is thought to inhibit angiogenesis through phosphorylation of SMAD2/3 11, which leads to increased expression of PAI‐1, a potent anti‐angiogenic factor 39. Our results confirm that ALK5 activation following cigarette smoke exposure is associated with increased expression of both phospho‐SMAD2/3 and PAI‐1. By contrast, phospho‐SMAD1/5, which is activated by ALK1, was not increased following exposure to cigarette smoke (Fig. 5). These results suggest that exposure to cigarette smoke modulates the TGF‐βR1 response, shifting the balance towards the anti‐angiogenic ALK5 pathway. The role of let‐7f in this pathophysiology was suggested by showing that let‐7f supplementation *in vitro* and *in vivo* could reduce the expression of ALK5, SMAD2/3 and PAI‐1 and that this was associated with improved angiogenesis and neovascularization in the context of cigarette smoke exposure. Moreover, the potential importance of the ALK5 pathway is illustrated by the fact that a direct inhibitor of ALK5 can restore the angiogenic activities (network formation, wound healing) of endothelial cells that are exposed to CSE. It is important to recognize however that miRNAs may have dozens or even hundreds of targets. Therefore, our study does not rule out the possibility that apart from ALK5, other factors could contribute to the pro‐angiogenic effects of let‐7f in these conditions.

The results of the present study suggest that PACs could also be involved in the modulation of neovascularization by let‐7f. PACs have been shown to reach ischaemic tissues where they can improve neovascularization either directly by incorporating into new vessels, or more often indirectly through paracrine secretion of angiogenic growth factors 5. Here we found that both the number and the functional activities of PACs were reduced in MES. However, treatment with a let‐7f mimic following surgically induced hindlimb ischaemia could restore the number and the functional activities of PACs (adhesion, migration, proliferation) in MES. miRNAs are increasingly recognized as important regulators of stem and progenitor cells 40. Interestingly, reduced let‐7f expression was previously found to be involved in the impaired functional activities of diabetic bone marrow‐derived angiogenic cells *in vitro* 41. Reduced let‐7f expression was linked to increased levels of the anti‐angiogenic factor thrombospondin 2 (TSP‐2) in that study, although let‐7f did not directly interact with TSP‐2 mRNA 41. Similarly to what we found in mature endothelial cells, it is possible that TGF‐β pathway is also involved in the modulation of PACs by let‐7f. Members of TGF‐β family have been shown to play important roles for self‐renewal, maintenance of pluripotency and differentiation of stem cells 42. Interestingly, it was recently demonstrated that the level of ALK1 increases whereas ALK5 decreases during endothelial progenitor cell (EPC) to endothelial cell differentiation and EPC‐mediated neovascularization 43. Therefore, it is plausible that let‐7f improves PAC functions by reducing the expression of ALK5, an effect that could be impaired in pathological conditions such as cigarette smoke exposure. However, only an association between PAC number/function, Let‐7f and neovascularization was shown in the current study. It is probable that a combination of factors contributes to impair PACs following cigarette smoke exposure. For example, reduction of NO expression following cigarette smoke exposure 13 could contribute to PAC dysfunction, as eNOS/NO pathway was shown to have an essential role for the mobilization of PACs after ischaemia 44. It is also important to recognize that there is currently no 100% specific marker for the identification of PACs. For example, a combination of CD31 or CD34 with c‐Kit has previously been used to identify PACs/EPCs in mouse peripheral blood and bone marrow 45. Future studies are therefore needed to better define the role of Let‐7f and ALK5 for the modulation of PACs in different conditions.

In conclusion, our study demonstrates for the first time that reduced let‐7f expression contributes to inhibit angiogenesis and ischaemia‐induced neovascularization following cigarette smoke exposure. Reduced let‐7f expression is associated with increased activation of ALK5, downstream stimulation of the anti‐angiogenic SMAD2/3 and PAI‐1 pathway, together with impaired angiogenic activities of mature endothelial cells and PACs. Our results also suggest that overexpression of let‐7f using a miR mimic could constitute a novel therapeutic strategy to improve ischaemia‐induced neovascularization in pathological conditions.

## Conflicts of interest

The authors confirm that there are no conflict of interests.

## Supporting information


**Figure S1** Effect of cigarette smoke exposure on Let‐7f expression in non‐ischemic hindlimb muscles.

**Figure S2** Transfection efficiency of miRs in HUVECs and hindlimb muscles.

**Figure S3** Effect of Let‐7f inhibition on endothelial cell function.
