# Supplementary figures and images for: Reduced expression of let‐7f activates TGF‐β/ALK5 pathway and leads to impaired ischaemia‐induced neovascularization after cigarette smoke exposure
Source: J Cell Mol Med. 2017 Mar 27;21(9):2211–22. doi: 10.1111/jcmm.13144 (PMC5571564; doi:10.1111/jcmm.13144)

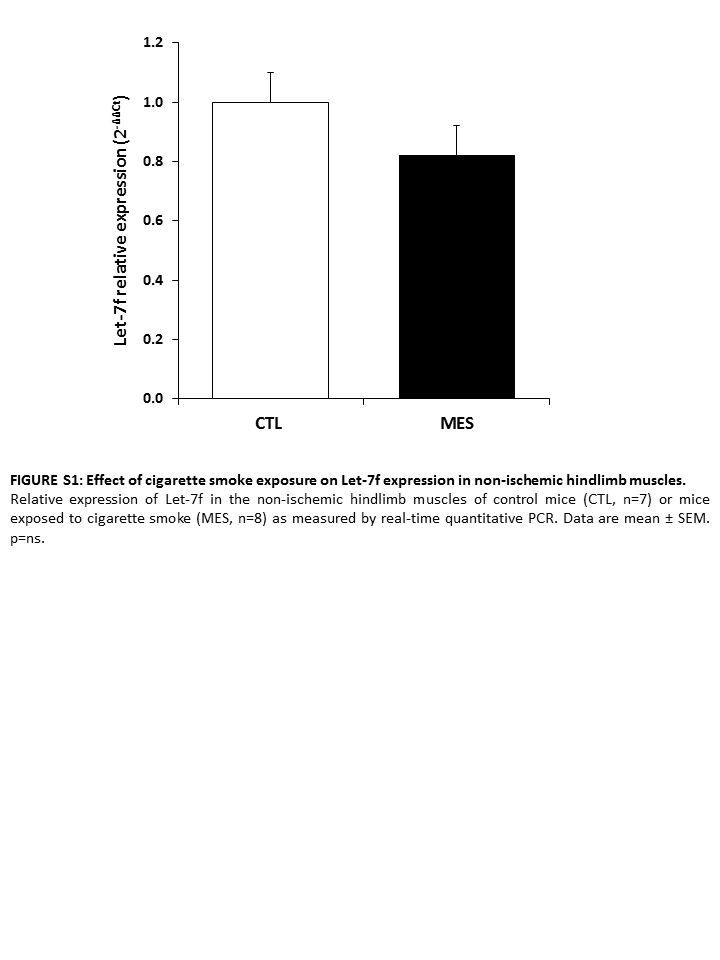

Supplement: Supplementary file 1 — Figure S1 Effect of cigarette smoke exposure on Let‐7f expression in non‐ischemic hindlimb muscles. [file JCMM-21-2211-s001.tif]

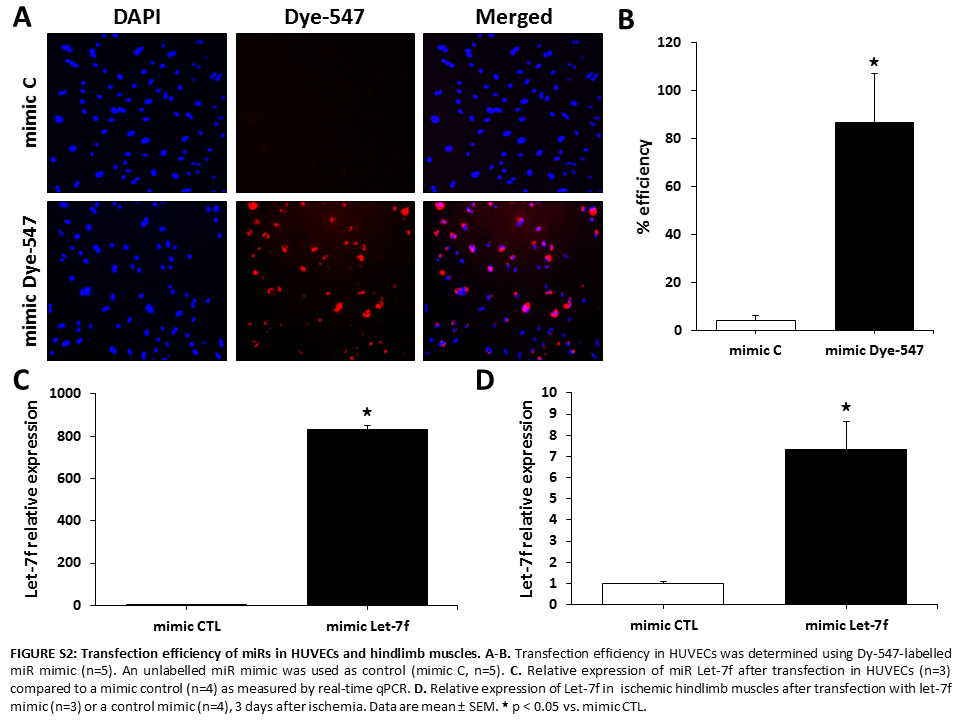

Supplement: Supplementary file 2 — Figure S2 Transfection efficiency of miRs in HUVECs and hindlimb muscles. [file JCMM-21-2211-s002.tif]

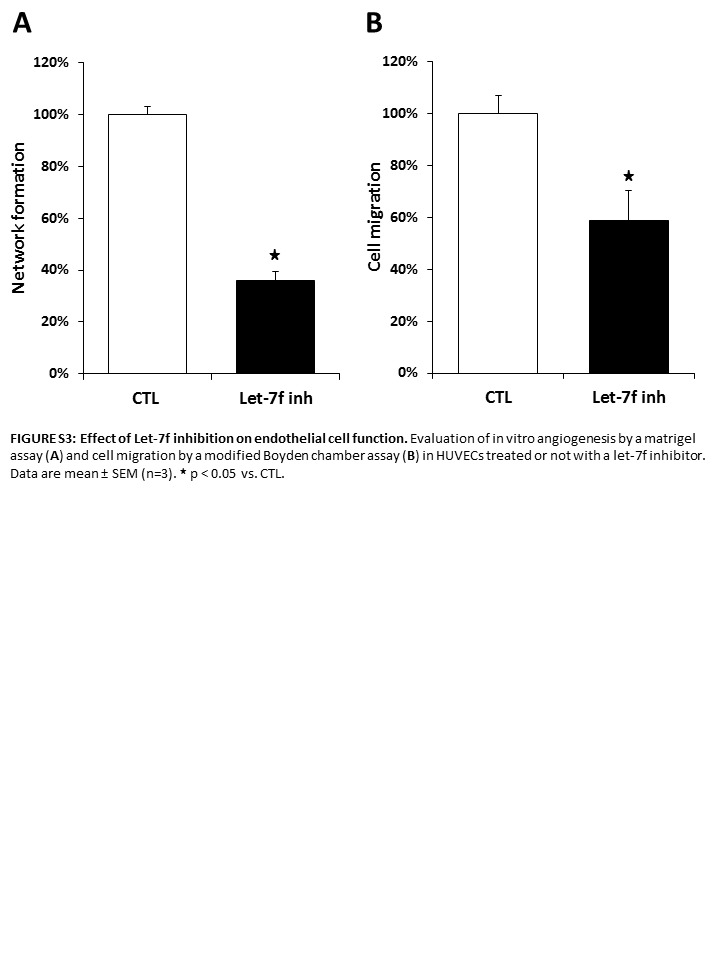

Supplement: Supplementary file 3 — Figure S3 Effect of Let‐7f inhibition on endothelial cell function. [file JCMM-21-2211-s003.tif]
